# *N*-(4-Methyl­benz­yl)-3-nitro­aniline

**DOI:** 10.1107/S1600536812024348

**Published:** 2012-05-31

**Authors:** Marijana Đaković, Tomislav Portada, Tin Klačić

**Affiliations:** a Department of Chemistry, Faculty of Science, University of Zagreb, Horvatovac 102a, HR-10000 Zagreb, Croatia; b Department of Organic Chemistry and Biochemistry, Ruder Bošković Institute, PO Box 180, HR-10000 Zagreb, Croatia; c 5th High School, Klaićeva 1, HR-10000 Zagreb, Croatia

## Abstract

In the title compound, C_14_H_14_N_2_O_2_, the angle between the mean plane of the *N*-methyl-3-nitro­aniline system (r.m.s. deviation = 0.0185 Å) and the *p*-tolyl unit is 89.79 (4)°. In the crystal, hydrogen-bonded chains running along [10-1] are generated by the linking of neighbouring mol­ecules *via* N—H⋯O and C—H⋯O hydrogen bonds involving the 3-nitro­aniline systems and forming *R*_2_^2^(8) motifs.

## Related literature

For related structures, see: Betz *et al.* (2011 ▶); Stilinović & Portada (2011 ▶); Xing *et al.* (2006 ▶). For the synthesis, see: Magyarfalvi (2008 ▶). For graph-set theory, see: Etter (1990 ▶); Bernstein *et al.* (1995 ▶).
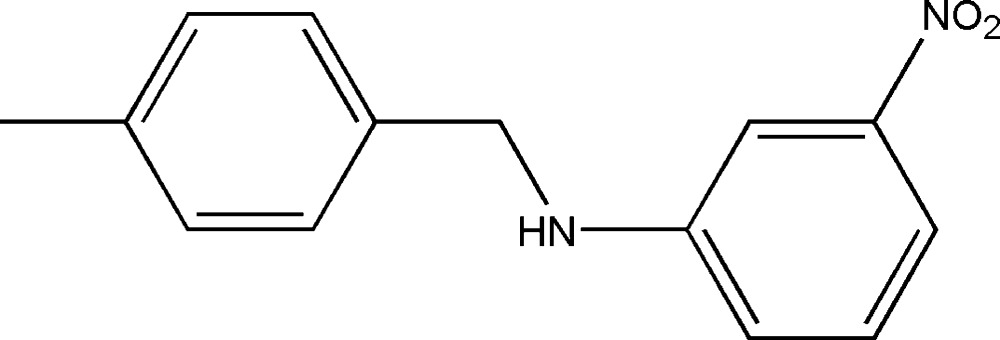


## Experimental

### 

#### Crystal data

C_14_H_14_N_2_O_2_
*M* *_r_* = 242.27
Monoclinic, 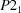

*a* = 5.1851 (4) Å
*b* = 21.408 (2) Å
*c* = 5.6833 (4) Å
β = 98.010 (7)°
*V* = 624.71 (8) Å^3^
*Z* = 2
Mo *K*α radiation
μ = 0.09 mm^−1^
*T* = 296 K
0.57 × 0.50 × 0.19 mm

#### Data collection

Oxford Diffraction Xcalibur diffractometer with a Sapphire-3 CCD area detector
Absorption correction: multi-scan (*CrysAlis PRO*; Oxford Diffraction, 2009 ▶) *T*_min_ = 0.953, *T*_max_ = 0.958
11868 measured reflections
1856 independent reflections
1373 reflections with *I* > 2σ(*I*)
*R*_int_ = 0.042

#### Refinement

*R*[*F* ^2^ > 2σ(*F* ^2^)] = 0.046
*wR*(*F* ^2^) = 0.117
*S* = 1.03
1856 reflections
167 parameters
1 restraint
H atoms treated by a mixture of independent and constrained refinement
Δρ_max_ = 0.16 e Å^−3^
Δρ_min_ = −0.11 e Å^−3^

### 

Data collection: *CrysAlis PRO* (Oxford Diffraction, 2009 ▶); cell refinement: *CrysAlis PRO*; data reduction: *CrysAlis PRO*; program(s) used to solve structure: *SHELXS97* (Sheldrick, 2008 ▶); program(s) used to refine structure: *SHELXL97* (Sheldrick, 2008 ▶); molecular graphics: *ORTEP-3* (Farrugia, 1997 ▶) and *Mercury* (Macrae *et al.*, 2006 ▶); software used to prepare material for publication: *SHELXL97* and *PLATON* (Spek, 2009 ▶).

## Supplementary Material

Crystal structure: contains datablock(s) global, I. DOI: 10.1107/S1600536812024348/fj2555sup1.cif

Structure factors: contains datablock(s) I. DOI: 10.1107/S1600536812024348/fj2555Isup2.hkl

Additional supplementary materials:  crystallographic information; 3D view; checkCIF report

## Figures and Tables

**Table 1:** Hydrogen-bond geometry (Å, °)

*D*—H⋯*A*	*D*—H	H⋯*A*	*D*⋯*A*	*D*—H⋯*A*
N1—H1*N*⋯O2^i^	0.78 (3)	2.52 (3)	3.277 (3)	168 (3)
C6—H6⋯O1^i^	0.93	2.44	3.364 (3)	171
C7—H7*A*⋯O2^ii^	0.97	2.64	3.352 (3)	130
C13—H13⋯O2^iii^	0.93	2.69	3.282 (4)	122

Symmetry codes: (i) 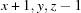; (ii) 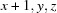; (iii) 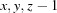.

**Fig. 1.**
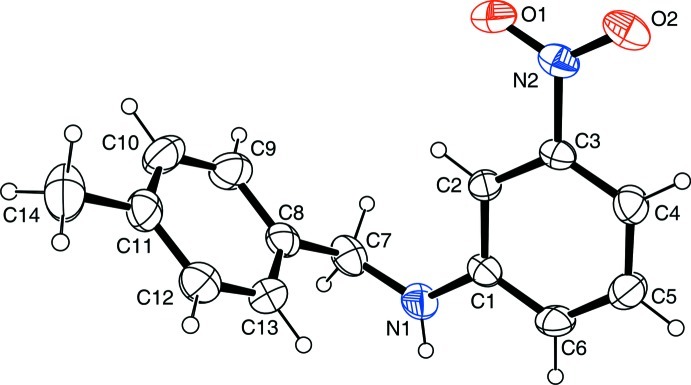
Molecular structure of the title compound with the atom labelling scheme. Displacement ellipsoids for non-H atoms are drawn at the 30% probability level. Hydrogen atoms are shown as a spheres of arbitrary radius.

**Fig. 2.**
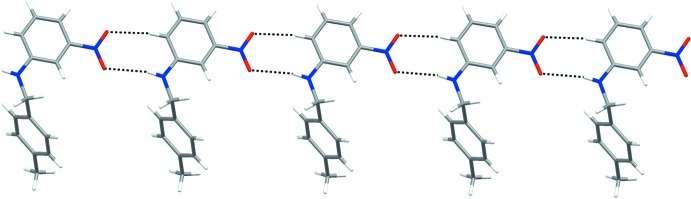
Infinite one-dimensional chains running in the [101] direction constructed via N—H···O and C—H···O hydrogen bonds between neighbouring molecules forming R22(8) motifs.

## supplementary crystallographic information

### Comment

The title compound, *N*-(4-methylbenzyl)-3-nitroaniline, is prepared as a part of the laboratory work with high school students, and the synthesis followed the Preparatory problems for the 40th International Chemistry Olympiad (Magyarfalvi, 2008) involving slight modifications.

Recently, *N*-benzyl-3-nitroaniline was reported (Stilinović & Portada, 2011). The difference between the title compound and the previously reported one is only in methyl substituent on the *N*-benzyl moiety, since it was of interest to study the influence of the benzyl moiety substituents on the molecular conformation, and consequently the hydrogen bonding formation.

The addition of methyl substituent on the benzyl moiety in the title compound did not cause any significant conformational difference. The molecule retained a bent conformation with the torsion angle about the central C—N bond of 73.9 (2)° being very similar to analogous one in the recently reported compound (Stilinović & Portada, 2011). Furthermore, the *N*-methyl-3-nitroaniline system in the title compound is nearly ideally planar (r.m.s. deviation of the atoms C1–C7/N1/N2/O1/O2 from their mean plane is 0.0185 Å, with oxygen atom O2 being the one that deviates most from that plane, 0.031 (2) Å). The *p*-tolyl substituent is tilted at an angle of 89.79 (4)° to the rest of the molecule.

Two neighbouring molecules are connected through the set of N—H···O and C—H···O hydrogen bonds in the head to tail manner forming *R*^2^_2_(8) motifs (Etter, 1990; Bernstein *et al.*, 1995) that generate one-dimensional chains running in the [101] direction. The same hydrogen bonding pattern is also found in *N*-benzyl-3-nitroaniline (Stilinović & Portada, 2011) what leads to the conclusion that the methyl substituent in *p*-position to the central C—N bond do not influence neither hydrogen bonding geometry nor general hydrogen bonding framework formation.

### Experimental

The title compound was prepared using a slightly modified procedure (Magyarfalvi, 2008) and isolated in a form of yellow crystalline product. Used: 3-nitroaniline (1.10 g; 7.96 mmol), *p*-tolualdehyde (1.74 ml; 1.77 g; 14.7 mmol), sodium tetrahydridoborate (0.50 g; 13.2 mmol). Yield: 1.12 g (58%). Upon re-crystallization in ethanol, yellow block-like crystalls suitable for the X-ray experiment were obtained in 3–4 days.

### Refinement

In the final cycles of refinement, in the absence of significant anomalous scattering effect, 1856 Friedel pairs were merged and Δf'' set to zero. The amine H atom was located in the difference Fourier map and freely refined, giving N—H distance of 0.78 (3) Å. All other H atoms were placed in geometrically idealized positions and constrained to ride on their parent C atom at distances of 0.93, 0.96 and 0.97 Å for aromatic, methyl and CH_2_ H atoms, respectively, and with *U*_iso_(H) = 1.2*U*_eq_(C) (for aromatic and CH_2_ H atoms), and *U*_iso_(H) = 1.5*U*_eq_(C) (for methyl group).

### Crystal data

**Table:**

C_14_H_14_N_2_O_2_	*F*(000) = 256
*M**_r_* = 242.27	*D*_x_ = 1.288 Mg m^−^^3^
Monoclinic, *P*2_1_	Mo *K*α radiation, λ = 0.71073 Å
Hall symbol: P 2 yb	Cell parameters from 4334 reflections
*a* = 5.1851 (4) Å	θ = 4.4–32.7°
*b* = 21.408 (2) Å	µ = 0.09 mm^−^^1^
*c* = 5.6833 (4) Å	*T* = 296 K
β = 98.010 (7)°	Plate, yellow
*V* = 624.71 (8) Å^3^	0.57 × 0.50 × 0.19 mm
*Z* = 2	

### Data collection

**Table:**

Oxford Diffraction Xcalibur diffractometer with a Sapphire-3 CCD area detector	1856 independent reflections
Radiation source: Enhance (Mo) X-ray Source	1373 reflections with *I* > 2σ(*I*)
Graphite monochromator	*R*_int_ = 0.042
Detector resolution: 16.3426 pixels mm^-1^	θ_max_ = 30.0°, θ_min_ = 4.4°
CCD scans	*h* = −7→7
Absorption correction: multi-scan (*CrysAlis PRO*; Oxford Diffraction, 2009)	*k* = −30→30
*T*_min_ = 0.953, *T*_max_ = 0.958	*l* = −7→7
11868 measured reflections	

### Refinement

**Table:**

Refinement on *F*^2^	Primary atom site location: structure-invariant direct methods
Least-squares matrix: full	Secondary atom site location: difference Fourier map
*R*[*F*^2^ > 2σ(*F*^2^)] = 0.046	Hydrogen site location: inferred from neighbouring sites
*wR*(*F*^2^) = 0.117	H atoms treated by a mixture of independent and constrained refinement
*S* = 1.03	*w* = 1/[σ^2^(*F*_o_^2^) + (0.0688*P*)^2^] where *P* = (*F*_o_^2^ + 2*F*_c_^2^)/3
1856 reflections	(Δ/σ)_max_ < 0.001
167 parameters	Δρ_max_ = 0.16 e Å^−^^3^
1 restraint	Δρ_min_ = −0.11 e Å^−^^3^

### Special details

**Table:**

Geometry. Bond distances, angles *etc*. have been calculated using the rounded fractional coordinates. All su's are estimated from the variances of the (full) variance-covariance matrix. The cell e.s.d.'s are taken into account in the estimation of distances, angles and torsion angles
Refinement. Refinement on *F*^2^ for ALL reflections except those flagged by the user for potential systematic errors. Weighted *R*-factors *wR* and all goodnesses of fit *S* are based on *F*^2^, conventional *R*-factors *R* are based on *F*, with *F* set to zero for negative *F*^2^. The observed criterion of *F*^2^ > σ(*F*^2^) is used only for calculating -*R*-factor-obs *etc*. and is not relevant to the choice of reflections for refinement. *R*-factors based on *F*^2^ are statistically about twice as large as those based on *F*, and *R*-factors based on ALL data will be even larger.

### Fractional atomic coordinates and isotropic or equivalent isotropic displacement parameters (Å^2^)

**Table:**

	*x*	*y*	*z*	*U*_iso_*/*U*_eq_	
O1	0.0400 (4)	0.59135 (11)	0.2460 (4)	0.0814 (8)	
O2	0.1836 (4)	0.49767 (11)	0.2911 (3)	0.0688 (7)	
N1	0.8389 (4)	0.47827 (11)	−0.2730 (5)	0.0645 (8)	
N2	0.1802 (4)	0.54885 (10)	0.1976 (3)	0.0525 (7)	
C1	0.6686 (4)	0.52300 (11)	−0.2149 (4)	0.0455 (6)	
C2	0.5065 (4)	0.51224 (10)	−0.0402 (4)	0.0417 (6)	
C3	0.3489 (4)	0.56021 (10)	0.0150 (4)	0.0434 (6)	
C4	0.3374 (5)	0.61797 (11)	−0.0925 (5)	0.0564 (8)	
C5	0.4936 (5)	0.62762 (13)	−0.2656 (5)	0.0627 (9)	
C6	0.6563 (5)	0.58119 (12)	−0.3245 (4)	0.0558 (8)	
C7	0.8676 (5)	0.41780 (13)	−0.1683 (5)	0.0619 (8)	
C8	0.6482 (4)	0.37253 (11)	−0.2473 (4)	0.0511 (7)	
C9	0.5891 (6)	0.32463 (15)	−0.1024 (5)	0.0703 (10)	
C10	0.3929 (7)	0.28214 (14)	−0.1763 (6)	0.0747 (11)	
C11	0.2506 (6)	0.28531 (12)	−0.3980 (5)	0.0628 (9)	
C12	0.3078 (6)	0.33344 (14)	−0.5412 (5)	0.0669 (9)	
C13	0.5021 (6)	0.37587 (13)	−0.4681 (5)	0.0608 (8)	
C14	0.0335 (7)	0.23991 (16)	−0.4795 (9)	0.0912 (13)	
H1N	0.913 (6)	0.4885 (14)	−0.376 (5)	0.061 (8)*	
H2	0.50580	0.47380	0.03580	0.0500*	
H4	0.22830	0.64920	−0.04980	0.0680*	
H5	0.48930	0.66590	−0.34350	0.0750*	
H6	0.76130	0.58890	−0.44120	0.0670*	
H7A	0.88320	0.42240	0.00290	0.0740*	
H7B	1.02900	0.39970	−0.20470	0.0740*	
H9	0.68320	0.32070	0.04860	0.0840*	
H10	0.35690	0.25070	−0.07280	0.0890*	
H12	0.21290	0.33750	−0.69180	0.0800*	
H13	0.53540	0.40770	−0.57100	0.0730*	
H14A	−0.04300	0.25020	−0.63840	0.1370*	
H14B	−0.09690	0.24240	−0.37530	0.1370*	
H14C	0.10230	0.19820	−0.47650	0.1370*	

### Atomic displacement parameters (Å^2^)

**Table:**

	*U*^11^	*U*^22^	*U*^33^	*U*^12^	*U*^13^	*U*^23^
O1	0.0779 (13)	0.0904 (15)	0.0858 (13)	0.0136 (12)	0.0468 (11)	−0.0109 (11)
O2	0.0736 (12)	0.0795 (13)	0.0590 (10)	−0.0050 (10)	0.0297 (9)	0.0073 (10)
N1	0.0566 (13)	0.0626 (13)	0.0834 (15)	−0.0065 (10)	0.0420 (12)	−0.0110 (11)
N2	0.0432 (10)	0.0693 (14)	0.0474 (10)	−0.0029 (9)	0.0144 (8)	−0.0086 (10)
C1	0.0394 (10)	0.0524 (12)	0.0462 (11)	−0.0081 (9)	0.0108 (8)	−0.0108 (9)
C2	0.0376 (9)	0.0446 (11)	0.0443 (10)	−0.0026 (8)	0.0111 (8)	−0.0001 (8)
C3	0.0394 (10)	0.0537 (12)	0.0387 (9)	−0.0031 (9)	0.0116 (8)	−0.0042 (8)
C4	0.0525 (13)	0.0498 (13)	0.0695 (14)	0.0056 (11)	0.0173 (11)	−0.0005 (12)
C5	0.0689 (16)	0.0546 (14)	0.0668 (15)	0.0009 (12)	0.0177 (13)	0.0136 (12)
C6	0.0534 (13)	0.0654 (15)	0.0530 (13)	−0.0136 (11)	0.0226 (10)	0.0013 (11)
C7	0.0446 (12)	0.0669 (15)	0.0753 (16)	0.0093 (12)	0.0120 (11)	−0.0171 (13)
C8	0.0521 (12)	0.0502 (12)	0.0528 (12)	0.0131 (10)	0.0142 (10)	−0.0040 (10)
C9	0.0801 (19)	0.0749 (17)	0.0551 (14)	0.0082 (16)	0.0069 (13)	0.0144 (14)
C10	0.085 (2)	0.0564 (15)	0.088 (2)	0.0077 (14)	0.0303 (17)	0.0262 (15)
C11	0.0629 (16)	0.0478 (13)	0.0820 (19)	0.0067 (11)	0.0252 (15)	−0.0065 (13)
C12	0.0719 (17)	0.0706 (17)	0.0572 (14)	−0.0026 (14)	0.0057 (12)	−0.0049 (13)
C13	0.0720 (15)	0.0569 (13)	0.0547 (13)	−0.0022 (13)	0.0135 (11)	0.0080 (12)
C14	0.078 (2)	0.0619 (18)	0.137 (3)	−0.0059 (15)	0.027 (2)	−0.021 (2)

### Geometric parameters (Å, º)

**Table:**

O1—N2	1.220 (3)	C11—C14	1.510 (5)
O2—N2	1.217 (3)	C11—C12	1.371 (4)
N1—C1	1.374 (3)	C12—C13	1.377 (4)
N1—C7	1.424 (4)	C2—H2	0.9300
N2—C3	1.468 (3)	C4—H4	0.9300
N1—H1N	0.78 (3)	C5—H5	0.9300
C1—C6	1.390 (3)	C6—H6	0.9300
C1—C2	1.406 (3)	C7—H7A	0.9700
C2—C3	1.376 (3)	C7—H7B	0.9700
C3—C4	1.377 (3)	C9—H9	0.9300
C4—C5	1.374 (4)	C10—H10	0.9300
C5—C6	1.375 (4)	C12—H12	0.9300
C7—C8	1.514 (4)	C13—H13	0.9300
C8—C13	1.374 (4)	C14—H14A	0.9600
C8—C9	1.376 (4)	C14—H14B	0.9600
C9—C10	1.386 (5)	C14—H14C	0.9600
C10—C11	1.370 (4)		
			
O1···C1^i^	3.362 (3)	C13···H7B^i^	3.0900
O1···C6^ii^	3.364 (3)	H1N···O2^vi^	2.52 (3)
O2···C7^i^	3.352 (3)	H1N···H6	2.3000
O2···C13^iii^	3.282 (4)	H2···O2	2.4200
O1···H14C^iv^	2.7900	H2···C7	2.6300
O1···H4	2.4000	H2···C8	2.8600
O1···H6^ii^	2.4400	H2···H7A	2.2800
O2···H1N^ii^	2.52 (3)	H4···O1	2.4000
O2···H2	2.4200	H5···H14C^viii^	2.5700
O2···H7A^i^	2.6400	H6···O1^vi^	2.4400
O2···H13^iii^	2.6900	H6···H1N	2.3000
N1···C3^v^	3.398 (3)	H6···H14C^viii^	2.5100
N2···C1^i^	3.333 (3)	H7A···O2^v^	2.6400
N1···H13	2.6200	H7A···C2	2.7300
C1···O1^v^	3.362 (3)	H7A···H2	2.2800
C1···N2^v^	3.333 (3)	H7A···H9	2.4400
C1···C13	3.520 (4)	H7B···C11^v^	2.9800
C2···C8	3.333 (3)	H7B···C12^v^	2.9200
C3···N1^i^	3.398 (3)	H7B···C13^v^	3.0900
C6···O1^vi^	3.364 (3)	H9···H7A	2.4400
C7···O2^v^	3.352 (3)	H9···H14A^ix^	2.6000
C8···C2	3.333 (3)	H12···H14A	2.3400
C13···C1	3.520 (4)	H13···O2^vii^	2.6900
C13···O2^vii^	3.282 (4)	H13···N1	2.6200
C2···H7A	2.7300	H14A···H9^x^	2.6000
C6···H14C^viii^	3.0800	H14A···H12	2.3400
C7···H2	2.6300	H14B···C9^i^	2.9800
C8···H2	2.8600	H14C···O1^xi^	2.7900
C9···H14B^v^	2.9800	H14C···C6^xii^	3.0800
C11···H7B^i^	2.9800	H14C···H5^xii^	2.5700
C12···H7B^i^	2.9200	H14C···H6^xii^	2.5100
			
C1—N1—C7	124.5 (2)	C1—C2—H2	121.00
O1—N2—O2	123.1 (2)	C3—C2—H2	121.00
O1—N2—C3	117.9 (2)	C3—C4—H4	121.00
O2—N2—C3	119.0 (2)	C5—C4—H4	121.00
C7—N1—H1N	123 (2)	C4—C5—H5	120.00
C1—N1—H1N	113 (2)	C6—C5—H5	120.00
N1—C1—C6	120.5 (2)	C1—C6—H6	119.00
C2—C1—C6	117.9 (2)	C5—C6—H6	119.00
N1—C1—C2	121.5 (2)	N1—C7—H7A	108.00
C1—C2—C3	118.1 (2)	N1—C7—H7B	108.00
N2—C3—C4	118.0 (2)	C8—C7—H7A	108.00
N2—C3—C2	117.97 (19)	C8—C7—H7B	108.00
C2—C3—C4	124.0 (2)	H7A—C7—H7B	107.00
C3—C4—C5	117.3 (2)	C8—C9—H9	119.00
C4—C5—C6	120.6 (2)	C10—C9—H9	119.00
C1—C6—C5	122.0 (2)	C9—C10—H10	119.00
N1—C7—C8	115.3 (2)	C11—C10—H10	119.00
C9—C8—C13	116.4 (2)	C11—C12—H12	119.00
C7—C8—C9	121.4 (2)	C13—C12—H12	119.00
C7—C8—C13	122.2 (2)	C8—C13—H13	119.00
C8—C9—C10	121.5 (3)	C12—C13—H13	119.00
C9—C10—C11	121.7 (3)	C11—C14—H14A	109.00
C10—C11—C14	122.2 (3)	C11—C14—H14B	110.00
C10—C11—C12	116.7 (3)	C11—C14—H14C	109.00
C12—C11—C14	121.0 (3)	H14A—C14—H14B	109.00
C11—C12—C13	121.8 (3)	H14A—C14—H14C	109.00
C8—C13—C12	121.9 (3)	H14B—C14—H14C	109.00
			
C7—N1—C1—C2	0.7 (4)	C3—C4—C5—C6	−0.9 (4)
C7—N1—C1—C6	179.2 (2)	C4—C5—C6—C1	0.6 (4)
C1—N1—C7—C8	73.9 (3)	N1—C7—C8—C9	−152.7 (3)
O1—N2—C3—C2	−179.8 (2)	N1—C7—C8—C13	29.0 (4)
O1—N2—C3—C4	−0.6 (3)	C7—C8—C13—C12	178.1 (3)
O2—N2—C3—C2	−0.8 (3)	C7—C8—C9—C10	−178.3 (3)
O2—N2—C3—C4	178.5 (2)	C13—C8—C9—C10	0.1 (4)
N1—C1—C2—C3	177.4 (2)	C9—C8—C13—C12	−0.3 (4)
C6—C1—C2—C3	−1.1 (3)	C8—C9—C10—C11	0.8 (5)
N1—C1—C6—C5	−178.1 (2)	C9—C10—C11—C12	−1.5 (5)
C2—C1—C6—C5	0.5 (4)	C9—C10—C11—C14	−179.0 (3)
C1—C2—C3—N2	−179.93 (19)	C10—C11—C12—C13	1.3 (5)
C1—C2—C3—C4	0.9 (3)	C14—C11—C12—C13	178.9 (3)
N2—C3—C4—C5	−179.1 (2)	C11—C12—C13—C8	−0.4 (5)
C2—C3—C4—C5	0.1 (4)		

Symmetry codes: (i) *x*−1, *y*, *z*; (ii) *x*−1, *y*, *z*+1; (iii) *x*, *y*, *z*+1; (iv) −*x*, *y*+1/2, −*z*; (v) *x*+1, *y*, *z*; (vi) *x*+1, *y*, *z*−1; (vii) *x*, *y*, *z*−1; (viii) −*x*+1, *y*+1/2, −*z*−1; (ix) *x*+1, *y*, *z*+1; (x) *x*−1, *y*, *z*−1; (xi) −*x*, *y*−1/2, −*z*; (xii) −*x*+1, *y*−1/2, −*z*−1.

### Hydrogen-bond geometry (Å, º)

**Table:**

*D*—H···*A*	*D*—H	H···*A*	*D*···*A*	*D*—H···*A*
N1—H1*N*···O2^vi^	0.78 (3)	2.52 (3)	3.277 (3)	168 (3)
C6—H6···O1^vi^	0.93	2.44	3.364 (3)	171
C7—H7*A*···O2^v^	0.97	2.64	3.352 (3)	130
C13—H13···O2^vii^	0.93	2.69	3.282 (4)	122

Symmetry codes: (v) *x*+1, *y*, *z*; (vi) *x*+1, *y*, *z*−1; (vii) *x*, *y*, *z*−1.
